# Lineshape of Amplitude-Modulated Stimulated Raman Spectra

**DOI:** 10.3390/s24216990

**Published:** 2024-10-30

**Authors:** Marco Lamperti, Lucile Rutkowski, Guglielmo Vesco, Luca Moretti, Davide Gatti, Giulio Cerullo, Dario Polli, Marco Marangoni

**Affiliations:** 1 Dipartimento di Scienza e Alta Tecnologia, Università Degli Studi dell’Insubria, 22100 Como, Italy; marco.lamperti@uninsubria.it; 2 University of Rennes, CNRS, IPR (Institut de Physique de Rennes) UMR 6251, F35000 Rennes, France; 3 Dipartimento di Fisica—Politecnico di Milano and IFN-CNR, Via Gaetano Previati 1/C, 23900 Lecco, Italy; guglielmo.vesco@polimi.it (G.V.);

**Keywords:** optical metrology, molecular hydrogen, Stimulated Raman Scattering, amplitude modulation, spectral lineshape

## Abstract

The amplitude modulation of a pump field and the phase-sensitive detection of a pump-induced intensity change of a probe field encompass a common practice in nonlinear spectroscopies to enhance the detection sensitivity. A drawback of this approach arises when the modulation frequency is comparable to the width of the spectral feature of interest, since the presence of sidebands in the amplitude-modulated pump field provides distortion to the observed spectral lineshape. This represents a problem when accurate measurements of spectral lineshapes and line positions are pursued, as recently happened in our group with the metrology of the Q(1) line in the 1-0 band of molecular hydrogen. The measurement was performed with a Stimulated Raman Scattering spectrometer that was calibrated, for the first time, against an optical frequency comb. In this work, we develop an analytical tool for nonlinear Stimulated Raman spectroscopies that allows us to precisely quantify spectral distortions arising from high-frequency amplitude modulation in one of the interacting fields. Once they are known, spectral distortions can be deconvolved from the measured spectra to retrieve unbiased data. The application of this tool to the measured spectra is discussed.

## 1. Introduction

A cornerstone of high-resolution molecular spectroscopy is the precise determination of line positions and shapes [1]. This subject has received renewed interest in the past decade to address fundamental questions related to the physics of molecular hydrogen [2,3,4], whose simple structure allows for very accurate theoretical calculations of its energy levels [5,6]. In comparing theoretical with experimental line-center frequencies, the knowledge of spectral lineshapes has often emerged as a limiting factor for the uncertainty budget and, thus, for the strength of the comparison. This happened both for Doppler-broadened lines observed in a strongly collisional environment [7,8,9,10,11,12,13,14] and for sub-Doppler lines measured at low pressure in molecular beams [15,16,17] or in optical cavities [18,19,20,21]. Actually, the quest for high precision and for a proper modeling of spectral profiles is very general and involves many other contexts, such as tests of quantum-electro-dynamics in molecular ions [22], determination of dissociation energies [23,24], measurements of fundamental physics constants [25] or of their possible temporal variation [26,27,28], search for parity violations [29,30] and primary thermometry [31,32,33]. This range of applications covers all spectral ranges, from microwave and submillimeter [34] wave spectroscopy to extreme ultraviolet spectroscopy [35].

A first step in any precision spectroscopy investigation is to access reliable spectral data by deconvolving experimental spectra from any broadening or distortion induced by the adopted apparatus and/or measurement approach. Several experimental conditions may affect the measured spectra, such as the laser emission linewidth [36], the adoption of modulation techniques [37] like wavelength modulation spectroscopy [38] or frequency modulation spectroscopy [39], the perturbation given by an optical cavity [40], or even the choice of the data sampling grid, as recently shown, for instance, with the interferograms produced by optical frequency combs in Fourier-transform spectrometers [41]. The correct modelling of any measurement-related distortion is a prerequisite to access spectra only dominated by the physics of the observed spectroscopic target. This problem came recently to relevance in our group with the development of a Stimulated Raman Loss (SRL) spectrometer for the metrology of quadrupole lines [42], which enabled us to measure the line position of the Q(1) 1-0 line of molecular hydrogen with a state-of-the-art ppb level uncertainty [43]. In this apparatus, to achieve shot-noise SRL spectra, we intensity modulate the Stokes field at high frequency and detect the modulation transfer to the pump field using a lock-in amplifier. This approach, due to a modulation frequency that is comparable to the width of the transition observed, results in a spectral distortion because of the sidebands associated with the amplitude modulation of the Stokes field.

In this work, we develop an analytical tool to model the shape of the observed SRL spectra. To this purpose, we integrate an amplitude-modulated Stokes field in the nonlinear propagation equations that describe Stimulated Raman Scattering with the associated sidebands and a frequency-dependent third-order nonlinear susceptibility to account for the sharp Raman resonance. The phase-sensitive detection of the SRL signal is also analytically modeled, to predict both the in-phase and quadrature signals at the output of the lock-in amplifier. The spectral shape of the quadrature signal, in particular, is found to be a useful indicator for the proper demodulation phase, while the shape of the in-phase signal can be used to deconvolve the measured data by any modulation-induced artifact. We discuss these artifacts in relation to our metrological investigation of H_2_, and we observe that the developed model sheds light to the origin of a peculiar spectral behavior for the quadrature signal observed years ago in a similar spectrometer [44], which the authors were not able to explain. As Supplementary Materials, we discuss the extent to which the phase-matching condition is satisfied in a Stimulated Raman Scattering process under amplitude modulation.

## 2. Lineshape Analysis in a Precision-Coherent Raman Spectrometer

The retrieval of the Raman lineshape net of any instrumental distortion is crucial in a metrological apparatus to study collisional effects and determine transition energies with high accuracy. In our case, a distortion arises because of a modulation frequency adopted for the Stokes beam (10 MHz) that is comparable to the linewidth of the target Raman transition; in the case of the studied Q(1) 1-0 line of H_2_ [43], the ratio is as small as 25 at a pressure of 2 atm, where the Raman response assumes a nearly Lorentzian profile with a width shrunk to 250 MHz due to the Dicke effect. Following a procedure firstly introduced by Rosasco for phase-modulated Stimulated Raman Scattering [45], we derive and discuss analytical formulas to quantify first- and second-order corrections to the measured SRL spectral response. The derivation is preceded by introductory paragraphs on the notation used for the third-order nonlinear susceptibility of the medium and on the nonlinear propagation equations adopted for the calculation of SRL.

### 2.1. Coherent Raman Processes and $\chi^{3}$ Notation

**Figure 1:**
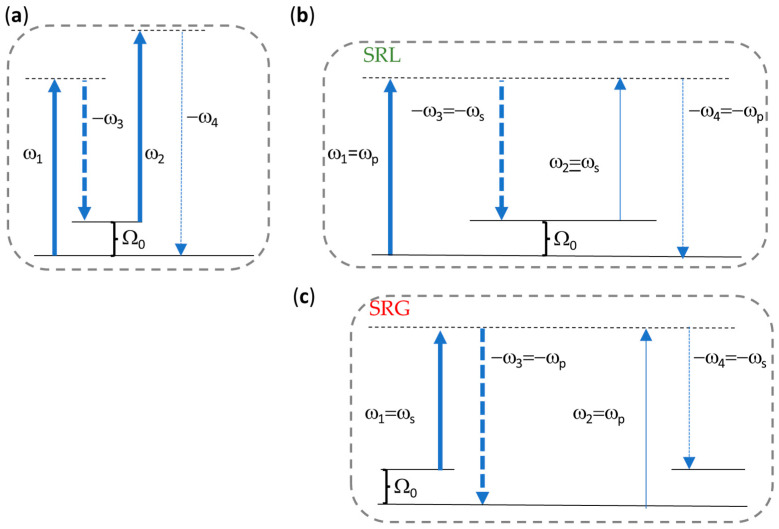
Solid arrows refer to absorption (photon annihilation), dashed lines to emission processes (photon generation). Bold arrows refer to the excitation fields, namely pump and Stokes fields (and also probe field in the general scheme in the (**a**) panel), whereas the grey frame encompasses the fields that are responsible for the nonlinear polarization of the medium. Dashed horizontal lines refer to the virtual energy levels through which the third-order nonlinear interaction takes place. Panels (**b**,**c**) are the photon energy diagrams in the degenerate case of Stimulated Raman Scattering, for an SRL and an SRG measurement, respectively.

Coherent Raman Scattering (CRS) identifies a class of third-order nonlinear optical spectroscopies that exploit two driving laser fields at distinct frequencies $\omega_{1}$ and $\omega_{3}$ to induce a vibrational coherence within an ensemble of irradiated molecules [46] and a third field at frequency $\omega_{2}$ to read out this coherence. The driving field with higher frequency is called pump ($\omega_{p}$), while that with lower frequency is called Stokes ($\omega_{s}$ < $\omega_{p}$). For a molecular vibration at frequency $\Omega_{0}$ to be resonantly excited through the third-order nonlinear susceptibility $\chi^{3}$ of a medium, the difference between pump and Stokes frequencies, namely $\omega_{p}-\omega_{s}$, must equal $\Omega_{0}$. The excitation can be detected in different ways. In the general interaction scheme shown in Figure 1a, for instance, where a probe field at a distinct frequency $\omega_{2}$ is also present, the excitation is encoded in the nonlinear emission of a fourth field at frequency ${\omega_{4}=\omega }_{1}-\omega_{3}+\omega_{2}$ that satisfies energy conservation. This is the so-called Coherent Antistokes Raman Scattering (CARS) process. In the absence of a probe field, that is, in a degenerate case, the process involved is Stimulated Raman Scattering (SRS). In this case, the measured nonlinear signal takes the form of either an attenuation of the pump field (Figure 1b), the so-called Stimulated Raman Loss (SRL), or in the form of an amplification of the Stokes field (Figure 1c), corresponding to the Stimulated Raman Gain (SRG). SRL and SRG are coexisting signals, and the convenience to detect one or the other depends on the specific experimental condition [47].

In the following, we will focus on SRS under the irradiance of pump and Stokes laser fields with parallel polarizations. This configuration allows us to exploit the largest coefficient of the third-order nonlinear susceptibility tensor, which is the diagonal $\chi_{1111}^{3}$ term [48]. For the sake of simplicity, we will refer to this term as $\chi^{3}$. Before entering an analytical description of the nonlinear interaction, we point out that the resonant excitation condition that underpins any CRS process implies a dependence of $\chi^{3}$ on the frequencies of the interacting fields. Following [45], we will express $\chi^{3}$ as $\chi \left(-\omega_{4},\omega_{1}{,\omega_{2},-\omega }_{3}\right)$, using a positive sign for the frequencies of the annihilated photons (up arrows in Figure 1) and a negative sign for those of created photons (down arrows), in such a way that:

- $\omega_{1}$ and $-\omega_{3}$ are the frequencies of the input pump and Stokes fields (bold arrows in Figure 1), obeying the relation $\left|\omega_{1}-\omega_{3}\right|=\omega_{p}-\omega_{s}$. In the SRS case, following the energy photon diagrams in Figure 1b and Figure 1c, respectively, one has $\omega_{1}\;$=${\;\omega }_{p}$ and $\omega_{3}\;$=${\;\omega }_{s}$ if the measured quantity is SRL, $\omega_{1}\;$=$\;\omega_{s}$ and $\omega_{3}\;$=$\;\omega_{p}$ for SRG measurements.
- $\omega_{2}$ is the frequency of the probe (read-out) field, corresponding to a bold up arrow in Figure 1a, where three distinct fields are considered at the input of the medium, and to a thin up arrow in the other panels where the probe field is degenerate with either the Stokes at $\omega_{s}$ (SRL case, Figure 1b) or the pump at $\omega_{p}$ (SRG case, Figure 1c).
$-\omega_{4}$ is the frequency of the field produced by the nonlinear polarization, which is equal to $-\omega_{p}$ and ${-\omega }_{s}$ for SRL and SRG, respectively.

According to the above convention, the nonlinear susceptibility involved in SRS processes reduces to $\chi^{3}=\chi \left(-\omega_{p},\omega_{p},\omega_{s}{,-\omega }_{s}\right)$ for SRL and $\chi^{3}=\chi \left(-\omega_{s},\omega_{s},\omega_{p}{,-\omega }_{p}\right)$ for SRG. The nonlinear polarization at $\omega_{4}$ takes the form $P_{NL}\propto {\chi^{3}E}_{1}E_{3}^{*}E_{2}$, where $E_{1}$ $E_{2}$ $E_{3}$ are the complex amplitudes of the electric fields at frequencies $\omega_{1},\omega_{2},\omega_{3}$, respectively. Due to degeneracy, one has $\omega_{4}=\omega_{p}$ and $P_{NL}\propto {\chi^{3}E}_{p}E_{s}^{*}E_{s}\propto {\chi^{3}I_{s}E}_{p}$ for the SRL case, $\omega_{4}=\omega_{s}$ and $P_{NL}\propto {\chi^{3}E}_{p}E_{p}^{*}E_{s}\propto {\chi^{3}I_{p}E}_{s}$ for the SRG case.

### 2.2. Nonlinear Propagation Equation in an SRS Process

From nonlinear optics textbooks [46,47], three fields at frequencies $\omega_{1}$, $\omega_{2},\omega_{3}$ propagating in a third-order nonlinear medium are responsible for a nonlinear polarization wave of the form:

$${\tilde{P}}_{NL}=\frac{1}{2}P_{NL}e^{i\left[\omega_{4}t-\left(k_{1}{-k}_{3}+k_{2}\right)z\right]}+cc$$

where $P_{NL}$ is its complex amplitude, ${\omega_{4}=\omega }_{1}-\omega_{3}+\omega_{2}$ is its oscillating frequency, $k_{1}$, $k_{2},k_{3}$ are the wavenumbers of the interacting electromagnetic fields. In a plane wave approximation, the field at frequency $\omega_{4}$ generated throughout the medium

$${\tilde{E}}_{4}=\frac{1}{2}E_{4}e^{i\left(\omega_{4}t-k_{4}z\right)}+cc$$

relates to the driving polarization wave via the equation:

(1) $$\frac{𝜕E_{4}}{𝜕z}=-\frac{i\mu_{0}\omega_{4}c}{2n_{4}}P_{NL}e^{i\left[k_{4}-\left(k_{1}{-k}_{3}+k_{2}\right)\right]z}$$

where z is the propagation coordinate, c is the speed of light, $\mu_{0}$ is the magnetic permeability, $n_{4}$ is the refractive index at $\omega_{4}$. The phase term on the righthand side of Equation (1) is affected by the phase mismatch $∆k=k_{4}-\left(k_{1}-k_{3}+k_{2}\right)$ between the driving and generated waves. In CARS processes, this term is often not negligible and eventually limits the interaction length and the amplitude of the generated nonlinear field. Differently, in an SRS process, the degeneracy condition $\omega_{1}=\omega_{4}=\omega_{p}$ and $\omega_{2}=\omega_{3}=\omega_{s}$ makes ∆k=0 and the interaction length only limited by the the interaction length only limited by the geometrical length of the sample. For the case of SRL that is of interest here, Equation (1) takes the simple form:

(2) $$\frac{𝜕E_{p}}{𝜕z}=-\frac{i\mu_{0}\omega_{p}c}{2n_{p}}P_{NL}$$

The solution of Equation (2) under intensity modulation of the Stokes field and, thus, in the presence of optical sidebands is the subject of the next paragraph. In typical treatments, the Stokes field is considered monochromatic because, in condensed matter, the spacing between the sidebands (MHz level) is orders of magnitude smaller than the probed vibrational resonances (hundreds GHz). Differently, when SRS is applied to molecular gases under certain pressure conditions, the spectral width of the vibrational resonance, and, therefore, of the observed SRS spectral response, may be comparable (hundreds MHz) to the sideband spacing. For precision spectroscopy, it is then mandatory to quantify the amount of spectral distortion caused by intensity modulation, as described in the following paragraph. Moreover, as the presence of sidebands affects the frequency degeneracy of an SRS process, a question also arises whether phase matching remains satisfied or not indefinitely, or at least over an experimental interaction length dictated by, e.g., a multi-pass cell; this will be a subject of discussion in the Supplementary Materials.

### 2.3. Generalized SRS Response under an Intensity-Modulated Excitation Field (SRL Case)

Let us consider an SRS process where the amplitude of the Stokes field is periodically modulated at an angular frequency $\omega_{m}$. The real part of this field can be expressed as follows:

(3) $${\tilde{E}}_{s}=\frac{1}{2}\left(E_{0}+E_{1}e^{i\omega_{m}t}+E_{-1}e^{-i\omega_{m}t}+E_{2}e^{2i\omega_{m}t}+E_{-2}e^{-2i\omega_{m}t}+\cdots \right)e^{i\left(\omega_{s}t-k_{s}z\right)}+c.c$$

with Fourier coefficients given by:

(4) $$E_{n}=\frac{1}{T}\int_{0}^{T}A\left(t\right)e^{-in\omega_{m}t}dt$$

where $T=2\pi /\omega_{m}\;$ is the modulation period and $A\left(t\right)$ is the periodic amplitude modulation function, which can be easily computed from the square root of the Stokes intensity profile $I_{s}\left(t\right)$. The different sidebands ${\tilde{E}}_{sn}$ that compose the Stokes field in Equation (3)

(5) $${\tilde{E}}_{s}={\tilde{E}}_{s0}+{\tilde{E}}_{s1}+{\tilde{E}}_{s-1}+{\tilde{E}}_{s2}+{\tilde{E}}_{s-2}+\ldots$$

can be expressed as:

(6) $$\begin{array}{c} {\tilde{E}}_{s0}=\frac{1}{2}E_{0}e^{i\left(\omega_{s}t-k_{s}z\right)}+c.c.\\ {\tilde{E}}_{s1}=\frac{1}{2}\left(E_{1}e^{i\left[\left(\omega_{s}+\omega_{m}\right)t-k_{s}z\right]}+c.c.\right)\\ {\tilde{E}}_{s-1}=\frac{1}{2}\left(E_{-1}e^{i\left[\left(\omega_{s}-\omega_{m}\right)t-k_{s}z\right]}+c.c.\right)\\ \ldots \ldots \end{array}$$

where the Fourier coefficients $E_{n}$ satisfy the Hermitian symmetry condition $E_{n}=E_{-n}^{*}$ because $A\left(t\right)$ is a real function. Let us now calculate the third-order nonlinear polarization in the presence of a monochromatic pump and a modulated Stokes field:

(7) $${\tilde{P}}_{NL}=\epsilon_{0}\chi^{3}{\left({\tilde{E}}_{p}+{\tilde{E}}_{s}\right)}^{3}$$

isolating the nonlinear polarization terms that oscillate at frequencies equal to either $\left(\omega_{p}-\omega_{m}\right)$ or $\left(\omega_{p}+\omega_{m}\right)$. These are indeed the only terms that, upon interference with the incoming pump field at $\omega_{p}$, may produce a beat note at $\omega_{m}$ to be synchronously detected with a lock-in amplifier, as for the measurement of SRL. Upon cubic expansion of Equation (7), the terms at $\left(\omega_{p}-\omega_{m}\right)$ happen to be:

(8) $$\begin{array}{c} {\tilde{P}}_{NL}\left(\omega_{p}-\omega_{m}\right)=\frac{3}{8}\epsilon_{0}\chi^{3}\left(-\left[\omega_{p}-\omega_{m}\right],\omega_{p},\left[\omega_{s}-\omega_{m}\right],-\omega_{s}\right)E_{p}E_{-1}E_{0}^{*}e^{i\left(\omega_{p}-\omega_{m}\right)t}+\\ +\frac{3}{8}\epsilon_{0}\chi^{3}\left(-\left[\omega_{p}-\omega_{m}\right],\omega_{p},\omega_{s},-\left[\omega_{s}+\omega_{m}\right]\right)E_{p}E_{0}E_{1}^{*}e^{i\left(\omega_{p}-\omega_{m}\right)t}+\\ +\frac{3}{8}\epsilon_{0}\chi^{3}\left(-\left[\omega_{p}-\omega_{m}\right],\omega_{p},\left[\omega_{s}-2\omega_{m}\right],-\left[\omega_{s}-\omega_{m}\right]\right)E_{p}E_{-2}E_{-1}^{*}e^{i\left(\omega_{p}-\omega_{m}\right)t}+\\ +\frac{3}{8}\epsilon_{0}\chi^{3}\left(-\left[\omega_{p}-\omega_{m}\right],\omega_{p},\left[\omega_{s}+\omega_{m}\right],-\left[\omega_{s}+{2\omega }_{m}\right]\right)E_{p}E_{1}E_{2}^{*}e^{i\left(\omega_{p}-\omega_{m}\right)t}+\\ +\frac{3}{8}\epsilon_{0}\chi^{3}\left(-\left[\omega_{p}-\omega_{m}\right],\omega_{p},\left[\omega_{s}-3\omega_{m}\right],-\left[\omega_{s}-{2\omega }_{m}\right]\right)E_{p}E_{-3}E_{-2}^{*}e^{i\left(\omega_{p}-\omega_{m}\right)t}+cc\\ +\frac{3}{8}\epsilon_{0}\chi^{3}\left(-\left[\omega_{p}-\omega_{m}\right],\omega_{p},\left[\omega_{s}+2\omega_{m}\right],-\left[\omega_{s}+{3\omega }_{m}\right]\right)E_{p}E_{2}E_{3}^{*}e^{i\left(\omega_{p}-\omega_{m}\right)t}+cc \end{array}$$

**Figure 2:**
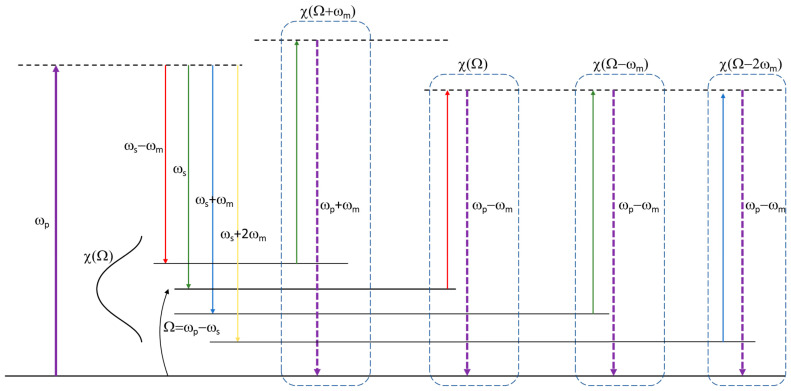
Different combinations of sidebands for the field at $\omega_{3}$ (experiencing stimulated emission from the virtual level after pump excitation) and $\omega_{2}$ (read-out field from the vibrational level) lead to the same emitted frequency $\omega_{4}$ (for example $\omega_{p}-\omega_{m}$ in the three different cases shown on the right of the figure); each combination corresponds to a different frequency detuning between $\omega_{1}=\omega_{p}$ and $\omega_{3}$ and thus to a different $\chi^{3}$ value. A case is also shown for an emitted field at frequency $\omega_{4}=\omega_{p}+\omega_{m}$.

Equation (8) shows that there are several combinations (namely products) of sidebands that lead to a nonlinear polarization oscillating at $\left(\omega_{p}-\omega_{m}\right)$, essentially all combinations where the readout frequency $\omega_{2}$ (third term in the $\chi^{3}$ bracket) is lower than the stimulated-emission frequency $\omega_{3}$ (fourth term in the $\chi^{3}$ bracket, sign convention apart) by $\omega_{m}$. The energy diagram reported in Figure 2 visualizes, as an example, three possible interactions that lead to a nonlinear polarization at $\omega_{4}=\left(\omega_{p}-\omega_{m}\right)$ and one interaction that leads to $\omega_{4}=\left(\omega_{p}+\omega_{m}\right)$. In Equation (8), we restricted the analysis to six different combinations, neglecting sidebands of order >3. The numeric coefficients 3 in front of any term in Equation (8) can be understood from the expansion of a trinomial cube: ${\left(a+b+c\right)}^{3}=a^{3}+b^{3}+c^{3}+3a^{2}b+3b^{2}a+3a^{2}c+3c^{2}a+3b^{2}c+3c^{2}b+6abc$ noticing that, for SRS, where just two fields are present, terms of the $a^{2}b$ kind are preceded by a 3 coefficient. In a more general case, with three distinct fields mixed up by the nonlinear response, the relevant coefficient is 6, as it precedes the mixed product abc (please note that one could also regard SRS as an interaction between three distinct fields, provided that two of them are taken identical and their amplitude rescaled by a factor of $\sqrt{2}$ in such a way to again obtain $\frac{6}{{\left(\sqrt{2}\right)}^{2}}=3$).

Looking more deeply at Equation (8), one recognizes that the six interactions considered, as mediated by different sideband orders, involve a different frequency detuning $\omega_{1}-\omega_{3}$: the first interaction, e.g., involves a frequency detuning $\omega_{p}-\omega_{s}=\Omega$, the second interaction a detuning $\omega_{p}-\left(\omega_{s}+\omega_{m}\right)=\Omega -\omega_{m}$, and so on (see also Figure 2). Accordingly, we may reshape Equation (8) in the compact form:

(9) $${\tilde{P}}_{NL}\left(\omega_{p}-\omega_{m}\right)=\frac{3}{8}\epsilon_{0}\left[\chi^{3}\left(\Omega \right)E_{-1}E_{0}^{*}+\chi^{3}\left(\Omega -\omega_{m}\right)E_{0}E_{1}^{*}+\chi^{3}\left(\Omega +\omega_{m}\right)E_{-2}E_{-1}^{*}\right]{E_{p}e}^{i\left(\omega_{p}-\omega_{m}\right)t}+\;\;+\frac{3}{8}\epsilon_{0}\left[\chi^{3}\left(\Omega -2\omega_{m}\right)E_{1}E_{2}^{*}+\chi^{3}\left(\Omega +{2\omega }_{m}\right)E_{-3}E_{-2}^{*}+\chi^{3}\left(\Omega -{3\omega }_{m}\right)E_{2}E_{3}^{*}\right]E_{p}e^{i\left(\omega_{p}-\omega_{m}\right)t}+cc$$

The $\chi^{3}\left(\Omega \pm \omega_{m}\right)$ and $\chi^{3}\left(\Omega \pm 2\omega_{m}\right)$ terms in Equation (9) are responsible for a distorted SRS spectral response, as long as a narrow Raman resonance (as occurs in a gas) prevents those terms to be considered constant across the modulated Stokes spectrum. They can be computed with a Taylor expansion of $\chi^{3}\;$ around the nominal frequency detuning Ω:

(10) $$\begin{array}{c} \chi^{3}\left(\Omega \pm \omega_{m}\right)=\chi^{3}\left(\Omega \right)+\frac{𝜕\chi^{3}}{𝜕\Omega }\left(\pm \omega_{m}\right)+\frac{1}{2}\frac{{𝜕}^{2}\chi^{3}}{𝜕\Omega^{2}}{\left(\pm \omega_{m}\right)}^{2}+\ldots \\ \chi^{3}\left(\Omega \pm {2\omega }_{m}\right)=\chi^{3}\left(\Omega \right)+\frac{𝜕\chi^{3}}{𝜕\Omega }\left(\pm {2\omega }_{m}\right)+\frac{1}{2}\frac{{𝜕}^{2}\chi^{3}}{𝜕\Omega^{2}}{\left(\pm 2\omega_{m}\right)}^{2}+\ldots \\ \chi^{3}\left(\Omega \pm {3\omega }_{m}\right)=\chi^{3}\left(\Omega \right)+\frac{𝜕\chi^{3}}{𝜕\Omega }\left(\pm {3\omega }_{m}\right)+\frac{1}{2}\frac{{𝜕}^{2}\chi^{3}}{𝜕\Omega^{2}}{\left(\pm 3\omega_{m}\right)}^{2}+\ldots \end{array}$$

The substitution of Equation (10) into Equation (9) returns for the nonlinear polarization at $\left(\omega_{p}-\omega_{m}\right)$ a very compact expression:

(11) $${\tilde{P}}_{NL}\left(\omega_{p}-\omega_{m}\right)=\frac{3}{8}\epsilon_{0}\chi_{eff-}^{3}E_{0}^{2}E_{p}e^{i\left(\omega_{p}-\omega_{m}\right)t}+cc$$

provided an effective nonlinear susceptibility is defined as follows:

(12) $$\begin{array}{c} {\chi_{eff-}^{3}=\chi }^{3}\left(\Omega \right)\left[\alpha_{-1}\alpha_{0}^{*}+\alpha_{0}\alpha_{1}^{*}+\alpha_{-2}\alpha_{-1}^{*}+\alpha_{1}\alpha_{2}^{*}+\alpha_{-3}\alpha_{-2}^{*}+\alpha_{2}\alpha_{3}^{*}\right]+\\ +\frac{𝜕\chi^{3}}{𝜕\Omega }\left[-\alpha_{0}\alpha_{1}^{*}+\alpha_{-2}\alpha_{-1}^{*}-2\alpha_{1}\alpha_{2}^{*}+{2\alpha }_{-3}\alpha_{-2}^{*}{-3\alpha }_{2}\alpha_{3}^{*}\right]\omega_{m}+\\ +\frac{1}{2}\frac{{𝜕}^{2}\chi^{3}}{𝜕\Omega^{2}}\left[\alpha_{0}\alpha_{1}^{*}+\alpha_{-2}\alpha_{-1}^{*}+4\alpha_{1}\alpha_{2}^{*}+{4\alpha }_{-3}\alpha_{-2}^{*}+9\alpha_{2}\alpha_{3}^{*}\right]\omega_{m}^{2} \end{array}$$

with complex coefficients $\alpha_{n}$ defined as $\alpha_{n}=\frac{E_{n}}{E_{0}}$. The effective nonlinear susceptibility $\chi_{eff-}^{3}\;$ depends not only on the nominal $\chi^{3}\left(\Omega \right)$ but also to its first- and second-order derivatives with respect to the frequency detuning Ω, through $\alpha_{i}\alpha_{j}^{*}$ weighting coefficients that depend on the specific intensity modulation waveform and also on the choice of the time axis origin ($\alpha_{i}\alpha_{j}^{*}$ are real for a time-symmetric waveform, for instance).

The same procedure followed so far can be repeated for the nonlinear polarization at frequency $\omega_{4}=\left(\omega_{p}+\omega_{m}\right)$:

(13) $$\begin{array}{c} {\tilde{P}}_{NL}\left(\omega_{p}+\omega_{m}\right)=\frac{3}{8}\epsilon_{0}\chi^{3}\left(-\left[\omega_{p}+\omega_{m}\right],\omega_{p},\omega_{s},-\left[\omega_{s}-\omega_{m}\right]\right)E_{p}E_{0}E_{-1}^{*}e^{i\left(\omega_{p}+\omega_{m}\right)t}+\\ \;+\frac{3}{8}\epsilon_{0}\chi^{3}\left(-\left[\omega_{p}+\omega_{m}\right],\omega_{p},{\left[\omega_{s}+\omega_{m}\right],-\omega }_{s},\right)E_{p}E_{1}E_{0}^{*}e^{i\left(\omega_{p}+\omega_{m}\right)t}+\\ \;+\frac{3}{8}\epsilon_{0}\chi^{3}\left(-\left[\omega_{p}+\omega_{m}\right],\omega_{p},\left[\omega_{s}-\omega_{m}\right],-\left[\omega_{s}-2\omega_{m}\right]\right)E_{p}E_{-1}E_{-2}^{*}e^{i\left(\omega_{p}+\omega_{m}\right)t}+\\ \;+\frac{3}{8}\epsilon_{0}\chi^{3}\left(-\left[\omega_{p}+\omega_{m}\right],\omega_{p},\left[\omega_{s}+{2\omega }_{m}\right],-\left[\omega_{s}+\omega_{m}\right]\right)E_{p}E_{2}E_{1}^{*}e^{+i\left(\omega_{p}+\omega_{m}\right)t}+\\ \;+\frac{3}{8}\epsilon_{0}\chi^{3}\left(-\left[\omega_{p}+\omega_{m}\right],\omega_{p},\left[\omega_{s}+{3\omega }_{m}\right],-\left[\omega_{s}+2\omega_{m}\right]\right)E_{p}E_{3}E_{2}^{*}e^{+i\left(\omega_{p}+\omega_{m}\right)t}+cc\\ +\frac{3}{8}\epsilon_{0}\chi^{3}\left(-\left[\omega_{p}+\omega_{m}\right],\omega_{p},\left[\omega_{s}-{2\omega }_{m}\right],-\left[\omega_{s}-3\omega_{m}\right]\right)E_{p}E_{-2}E_{-3}^{*}e^{+i\left(\omega_{p}+\omega_{m}\right)t}+cc \end{array}$$

which can be simplified to:

(14) $$\begin{array}{c} {\tilde{P}}_{NL}\left(\omega_{p}+\omega_{m}\right)=\frac{3}{8}\epsilon_{0}\left[\chi^{3}\left(\Omega +\omega_{m}\right)E_{0}E_{-1}^{*}+\chi^{3}\left(\Omega \right)E_{1}E_{0}^{*}+\chi^{3}\left(\Omega +2\omega_{m}\right)E_{-1}E_{-2}^{*}\right]{E_{p}e}^{i\left(\omega_{p}+\omega_{m}\right)t}+\\ +\frac{3}{8}\epsilon_{0}\left[\chi^{3}\left(\Omega -\omega_{m}\right)E_{2}E_{1}^{*}+\chi^{3}\left(\Omega -{2\omega }_{m}\right)E_{3}E_{2}^{*}+\chi^{3}\left(\Omega +{3\omega }_{m}\right)E_{-2}E_{-3}^{*}\right]E_{p}e^{i\left(\omega_{p}+\omega_{m}\right)t}+cc \end{array}$$

and set in the compact form:

(15) $${\tilde{P}}_{NL}\left(\omega_{p}+\omega_{m}\right)=\frac{3}{8}\epsilon_{0}\chi_{eff+}^{3}E_{0}^{2}E_{p}e^{i\left(\omega_{p}+\omega_{m}\right)t}+cc$$

by definition of an effective nonlinear susceptibility $\chi_{eff+}^{3}$:

(16) $$\begin{array}{c} {\chi_{eff+}^{3}=\chi }^{3}\left(\Omega \right)\left[\alpha_{0}\alpha_{-1}^{*}+\alpha_{1}\alpha_{0}^{*}+\alpha_{-1}\alpha_{-2}^{*}+\alpha_{2}\alpha_{1}^{*}+\alpha_{3}\alpha_{2}^{*}+\alpha_{-2}\alpha_{-3}^{*}\right]+\\ +\frac{𝜕\chi^{3}}{𝜕\Omega }\left[\alpha_{0}\alpha_{-1}^{*}+{2\alpha }_{-1}\alpha_{-2}^{*}-\alpha_{2}\alpha_{1}^{*}-2\alpha_{3}\alpha_{2}^{*}+3\alpha_{-2}\alpha_{-3}^{*}\right]\omega_{m}+\\ +\frac{1}{2}\frac{{𝜕}^{2}\chi^{3}}{𝜕\Omega^{2}}\left[\alpha_{0}\alpha_{-1}^{*}+{4\alpha }_{-1}\alpha_{-2}^{*}+\alpha_{2}\alpha_{1}^{*}+{4\alpha }_{3}\alpha_{2}^{*}+9\alpha_{-2}\alpha_{-3}^{*}\right]\omega_{m}^{2}+ \end{array}$$

By comparing Equations (12) and (16), one can notice that the weighting coefficients for $\chi^{3}\left(\Omega \right)$ and its first- and second-order perturbations have the same magnitude, yet not the same phase. Specifically, one may write:

(17) $$\begin{array}{c} {\chi_{eff-}^{3}=b_{0}\chi }^{3}\left(\Omega \right)+b_{1}\frac{𝜕\chi^{3}}{𝜕\Omega }\omega_{m}+b_{2}\frac{1}{2}\frac{{𝜕}^{2}\chi^{3}}{𝜕\Omega^{2}}\omega_{m}^{2}\\ {\chi_{eff-}^{3}=b_{0}^{*}\chi }^{3}\left(\Omega \right)-b_{1}^{*}\frac{𝜕\chi^{3}}{𝜕\Omega }\omega_{m}+b_{2}^{*}\frac{1}{2}\frac{{𝜕}^{2}\chi^{3}}{𝜕\Omega^{2}}\omega_{m}^{2} \end{array}$$

with:

(18) $$\begin{array}{c} b_{0}=\left[\alpha_{-1}\alpha_{0}^{*}+\alpha_{0}\alpha_{1}^{*}+\alpha_{-2}\alpha_{-1}^{*}+\alpha_{1}\alpha_{2}^{*}+\alpha_{-3}\alpha_{-2}^{*}+\alpha_{2}\alpha_{3}^{*}\right]\\ b_{1}=\left[-\alpha_{0}\alpha_{1}^{*}+\alpha_{-2}\alpha_{-1}^{*}-2\alpha_{1}\alpha_{2}^{*}+{2\alpha }_{-3}\alpha_{-2}^{*}{-3\alpha }_{2}\alpha_{3}^{*}\right]\\ b_{2}=\left[\alpha_{0}\alpha_{1}^{*}+\alpha_{-2}\alpha_{-1}^{*}+4\alpha_{1}\alpha_{2}^{*}+{4\alpha }_{-3}\alpha_{-2}^{*}+9\alpha_{2}\alpha_{3}^{*}\right] \end{array}$$

and:

(19) $$b_{0}+2b_{1}=0$$

We are now in a position to write the differential nonlinear propagation Equation (1) for the forced fields $E_{-}$ and $E_{+}$, which are, respectively, generated by the nonlinear polarization waves at $\left(\omega_{p}-\omega_{m}\right)$ and $\left(\omega_{p}+\omega_{m}\right)$:

(20) $$\begin{array}{c} \frac{𝜕E_{-}}{𝜕z}=-\frac{i\mu_{0}\left(\omega_{p}-\omega_{m}\right)c}{2n_{p}}P_{NL}\left(\omega_{p}-\omega_{m}\right)\approx -\frac{i\mu_{0}\omega_{p}c}{2n_{p}}P_{NL}\left(\omega_{p}-\omega_{m}\right)\\ \frac{𝜕E_{+}}{𝜕z}=-\frac{i\mu_{0}\left(\omega_{p}+\omega_{m}\right)c}{2n_{p}}P_{NL}\left(\omega_{p}+\omega_{m}\right)\approx -\frac{i\mu_{0}\omega_{p}c}{2n_{p}}P_{NL}\left(\omega_{p}+\omega_{m}\right) \end{array}$$

With the help of Equation (11) and Equation (15), Equation (20) becomes:

(21) $$\begin{array}{c} \frac{𝜕E_{-}}{𝜕z}=-\frac{i\mu_{0}\omega_{p}c}{2n_{p}}\frac{3}{4}\epsilon_{0}\chi_{eff-}^{3}E_{p}E_{0}^{2}=-\frac{3}{8}\frac{i\omega_{p}}{{cn}_{p}}\chi_{eff-}^{3}E_{p}E_{0}^{2}\\ \frac{𝜕E_{+}}{𝜕z}=-\frac{i\mu_{0}\omega_{p}c}{2n_{p}}\frac{3}{4}\epsilon_{0}\chi_{eff\mp }^{3}E_{p}E_{0}^{2}=-\frac{3}{8}\frac{i\omega_{p}}{{cn}_{p}}\chi_{eff+}^{3}E_{p}E_{0}^{2} \end{array}$$

where the $\frac{3}{8}$ coefficients of Equations (11) and (15) have been replaced by $\frac{3}{4}$ since Equation (1) requires the complex amplitude $P_{NL}$ rather than the real part ${\tilde{P}}_{NL}$. The integration of Equation (21) over an interaction length L is straightforward, since no depletion occurs for pump and Stokes fields. We obtain:

(22) $$\begin{array}{c} {∆E}_{-}=-\frac{3}{8}\frac{i\omega_{p}}{{cn}_{p}}\chi_{eff-}^{3}E_{p}E_{0}^{2}L\\ {∆E}_{+}=-\frac{3}{8}\frac{i\omega_{p}}{{cn}_{p}}\chi_{eff+}^{3}E_{p}E_{0}^{2}L \end{array}$$

where ${∆E}_{-}$ and ${∆E}_{+}$ are the amplitudes of the generated nonlinear fields at the output of the medium that are responsible for the SRL. If, in Equation (22), we replace the effective nonlinear susceptibilities with the expressions in Equation (17) and we add the time oscillation term that is the relevant for the lock-in detection, the overall forced (or nonlinearly generated) field is:

(23) $$\begin{array}{cc} ∆E= & -\frac{3}{8}\frac{i\omega_{p}}{{cn}_{p}}E_{p}E_{0}^{2}L[\left(b_{0}\chi^{3}\left(\Omega \right)+b_{1}\frac{𝜕\chi^{3}}{𝜕\Omega }\omega_{m}+b_{2}\frac{1}{2}\frac{{𝜕}^{2}\chi^{3}}{𝜕\Omega^{2}}\omega_{m}^{2}\right)e^{i\left(\omega_{p}-\omega_{m}\right)t}\\ & \;+\left(b_{0}^{*}\chi^{3}\left(\Omega \right)-b_{1}^{*}\frac{𝜕\chi^{3}}{𝜕\Omega }\omega_{m}+b_{2}^{*}\frac{1}{2}\frac{{𝜕}^{2}\chi^{3}}{𝜕\Omega^{2}}\omega_{m}^{2}\right)e^{i\left(\omega_{p}+\omega_{m}\right)t}]\\ & \;=\left[\left|{∆E}_{0}^{-}\right|e^{i\varphi_{0}^{-}}+\left|{∆E}_{1}^{-}\right|e^{i\varphi_{1}^{-}}+\left|{∆E}_{2}^{-}\right|e^{i\varphi_{2}^{-}}\right]e^{i\left(\omega_{p}-\omega_{m}\right)t}\\ & \;+\left[\left|{∆E}_{0}^{\mp }\right|e^{i\varphi_{0}^{+}}+\left|{∆E}_{1}^{+}\right|e^{i\varphi_{1}^{+}}+\left|{∆E}_{2}^{+}\right|e^{i\varphi_{2}^{+}}\right]e^{i\left(\omega_{p}+\omega_{m}\right)t} \end{array}$$

where the amplitude $\left|{∆E}_{i}^{\pm }\right|$ and phase $\varphi_{i}^{\pm }$ of any susceptibility order *i* have been highlighted, according to the definitions:

(24) $$\begin{array}{c} \left|{∆E}_{0}^{\pm }\right|=\frac{3}{8}\frac{\omega_{p}}{{cn}_{p}}E_{p}E_{0}^{2}L\left|b_{0}\right|\left|\chi^{3}\left(\Omega \right)\right|,\varphi_{0}^{\pm }=-\frac{\pi }{2}\mp \varphi_{b0}+\varphi_{\chi }\\ \left|{∆E}_{1}^{-}\right|=\frac{3}{8}\frac{\omega_{p}}{{cn}_{p}}E_{p}E_{0}^{2}L\left|b_{1}\right|\left|\frac{𝜕\chi^{3}}{𝜕\Omega }\right|\omega_{m},\varphi_{1}^{-}=-\frac{\pi }{2}{+\varphi }_{b1}+\varphi_{\chi \prime }\\ \left|{∆E}_{1}^{+}\right|=\frac{3}{8}\frac{\omega_{p}}{{cn}_{p}}E_{p}E_{0}^{2}L\left|b_{1}\right|\left|\frac{𝜕\chi^{3}}{𝜕\Omega }\right|\omega_{m},\varphi_{1}^{+}=-\frac{\pi }{2}+\left({\pi -\varphi }_{b1}\right)+\varphi_{\chi \prime }\\ \left|{∆E}_{2}^{\pm }\right|=\frac{3}{8}\frac{\omega_{p}}{{cn}_{p}}E_{p}E_{0}^{2}L\left|b_{2}\right|\frac{1}{2}\left|\frac{{𝜕}^{2}\chi^{3}}{𝜕\Omega^{2}}\right|\omega_{m}^{2},\varphi_{2}^{\pm }=-\frac{\pi }{2}\mp \varphi_{b2}+\varphi_{\chi \prime \prime } \end{array}$$

with $\varphi_{\chi }=\arg\chi^{3}\left(\Omega \right)$, $\varphi_{\chi \prime }=\arg\frac{𝜕\chi^{3}}{𝜕\Omega }$ $\varphi_{\chi \prime \prime }=\arg\frac{{𝜕}^{2}\chi^{3}}{𝜕\Omega^{2}}$, and while the $-\frac{\pi }{2}$ phase comes from the negative imaginary unit.

The last step is the computation of the SRL that originates from the superposition on a photodetector of the nonlinear field ∆E with a pump field of the form $E_{p}e^{i\omega_{p}t}$: through interference, they are responsible for a beat note signal $I_{b}$ at $\omega_{m}$ (to be demodulated with a lock-in amplifier) that contains the vibrational signature of interest. The real part of $I_{b}$ may be written as:

(25) $$I_{b}=\left|{∆E}_{0}^{-}\right|E_{p}cos\left(\omega_{m}t-\varphi_{0}^{-}\right)+\left|{∆E}_{0}^{+}\right|E_{p}cos\left(\omega_{m}t+\varphi_{0}^{+}\right)+\left|{∆E}_{1}^{-}\right|E_{p}cos\left(\omega_{m}t-\varphi_{1}^{-}\right)+\left|{∆E}_{1}^{+}\right|E_{p}cos\left(\omega_{m}t+\varphi_{1}^{+}\right)\;+\left|{∆E}_{2}^{-}\right|E_{p}cos\left(\omega_{m}t-\varphi_{2}^{-}\right)+\left|{∆E}_{0}^{+}\right|E_{p}cos\left(\omega_{m}t+\varphi_{2}^{+}\right)$$

Equation (25) subtends the well-known calculation of the square of the sum of two interfering fields, ${\left[Acos\left(\omega_{a}t+\varphi_{a}\right)+Bcos\left(\omega_{b}t+\varphi_{b}\right)\right]}^{2}$, which leads to the mixed product $2ABcos\left(\omega_{a}t+\varphi_{a}\right)cos\left(\omega_{b}t+\varphi_{b}\right)$ and then, by Werner laws, to an irrelevant sum-frequency term far beyond the detector bandwidth and a relevant difference-frequency term $ABcos\left[\left(\omega_{a}-\omega_{b}\;\right)t+\left(\varphi_{a}-\varphi_{b}\;\right)\right]$ that falls within the detector bandwidth. Exploiting the identities $\left|{∆E}_{i}^{-}\right|=\left|{∆E}_{i}^{+}\right|=\left|∆E_{i}\right|$ and the trigonometric law $cos\alpha +cos\beta =2cos\left(\frac{\alpha +\beta }{2}\right)cos\left(\frac{\alpha -\beta }{2}\right)$, the beat note signal may be written as:

(26) $$I_{b}=\sum_{i=0}^{2}2\left|∆E_{i}\right|E_{p}cos\left(\frac{\varphi_{i}^{+}+\varphi_{i}^{-}}{2}\right)cos\left(\omega_{m}t+\frac{\varphi_{i}^{+}-\varphi_{i}^{-}}{2}\right)$$

whose demodulation, using a local oscillator with an adjustable phase ∆,

(27) $$I_{d}=\frac{1}{\pi }\int_{-\pi }^{\pi }\left[\sum_{i=0}^{2}2\left|∆E_{i}\right|E_{p}cos\left(\frac{\varphi_{i}^{+}+\varphi_{i}^{-}}{2}\right)cos\left(\omega_{m}t+\frac{\varphi_{i}^{+}-\varphi_{i}^{-}}{2}\right)\right]cos(\omega_{m}t\;-∆)d\left(\omega_{m}t\right)$$

returns the following lock-in output signal

(28) $$I_{d}=\frac{1}{\pi }2\pi \sum_{i=0}^{2}\left|∆E_{i}\right|E_{p}cos\left(\frac{\varphi_{i}^{-}+\varphi_{i}^{+}}{2}\right)cos\left(\frac{\varphi_{i}^{+}-\varphi_{i}^{-}}{2}+∆\right)$$

which can be usefully expanded by separating the 0th-order term, which is related to $\chi^{3}\left(\Omega \right)$, from the first- and second-order terms related to its derivatives:

(29) $$\begin{array}{c} I_{d0}=2\left|∆E_{0}\right|E_{p}cos\left(\varphi_{\chi }-\frac{\pi }{2}\right)cos\left(\varphi_{b0}-∆\right)=2\left|∆E_{0}\right|E_{p}sen\left(\varphi_{\chi }\right)cos\left(\varphi_{b0}-∆\right)\\ I_{d1}=2\left|∆E_{1}\right|E_{p}cos\left(\varphi_{\chi \prime }\right)cos\left(\varphi_{b1}-\frac{\pi }{2}-∆\right)=2\left|∆E_{1}\right|E_{p}cos\left(\varphi_{\chi \prime }\right)sen\left(\varphi_{b1}-∆\right)\\ I_{d2}=2\left|∆E_{2}\right|E_{p}cos\left(\varphi_{\chi \prime \prime }-\frac{\pi }{2}\right)cos\left(\varphi_{b2}-∆\right)=2\left|∆E_{2}\right|E_{p}sen\left(\varphi_{\chi \prime \prime }\right)cos\left(\varphi_{b2}-∆\right) \end{array}$$

By setting $∆=\varphi_{b0}$ in Equation (29), which is the optimal lock-in phase to extract the vibrational response, and taking advantage of the phase relationship $\varphi_{b1}\;$−${\;\varphi }_{b0}=\pi$ that comes from Equation (19), the Stimulated Raman Loss terms associated with the interaction can be eventually computed and related to the easy measurable average Stokes field intensity $\langle I_{s}\rangle =\frac{1}{2}\epsilon_{0}cn_{s}E_{0}^{2}$:

(30) $$\begin{array}{c} SRL_{0}=\frac{I_{d0}}{E_{p}^{2}}=\frac{1}{E_{p}^{2}}2\left|∆E_{0}\right|E_{p}sen\left(\varphi_{\chi }\right)cos\left(\pi \right)=\frac{3}{2}\frac{\omega_{p}}{{{\varepsilon_{0}c}^{2}n}_{p}n_{s}}\langle I_{s}\rangle L\left|b_{0}\right|Im\left\{\chi^{3}\left(\Omega \right)\right\}\\ SRL_{1}=\frac{I_{d1}}{E_{p}^{2}}=\frac{1}{E_{p}^{2}}2\left|∆E_{1}\right|E_{p}cos\left(\varphi_{\chi \prime }\right)sen\left(\pi \right)\;\;=\frac{3}{2}\frac{\omega_{p}}{{{\varepsilon_{0}c}^{2}n}_{p}n_{s}}\langle I_{s}\rangle L\left|b_{1}\right|Re\left\{\frac{𝜕\chi^{3}}{𝜕\Omega }\right\}\omega_{m}sen\left(\pi \right)=0\\ SRL_{2}=\frac{I_{d2}}{E_{p}^{2}}=\frac{1}{E_{p}^{2}}2\left|∆E_{2}\right|E_{p}sen\left(\varphi_{\chi \prime \prime }\right)cos\left(\varphi_{b2}-\varphi_{b0}\right)\;\;=\frac{3}{2}\frac{\omega_{p}}{{{\varepsilon_{0}c}^{2}n}_{p}n_{s}}\langle I_{s}\rangle L\left|b_{2}\right|\frac{1}{2}Im\left\{\frac{{𝜕}^{2}\chi^{3}}{𝜕\Omega^{2}}\right\}\omega_{m}^{2}cos\left(\varphi_{b2}-\varphi_{b0}\right) \end{array}$$

**Figure 3:**
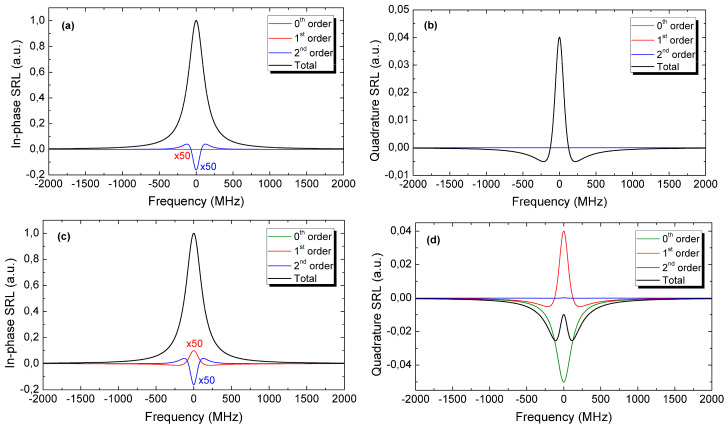
The curves shown in this figure refer to a 250-MHz Lorentzian Raman profile and to an intensity modulation waveform at 10 MHz that well fits that used in our experiments. Panels (**a**) and (**b**) report the normalized in-phase and quadrature SRL signals, respectively, when an optical demodulation phase (see text) is selected, while panels (**c**,**d**) report the same curves for a phase error of 0.05 rad (2.9°). Demodulation phase errors are responsible for the onset of a 1st order perturbation in the in-phase SRL signal (compare (**c**) vs. (**a**) panel) and for an evident change of the spectral response of the total quadrature signal, from a single- to a double-lobe curve (compare (**d**) vs. (**b**) panel). The lack of some orders in panels (**a**–**c**) is only apparent: depending on the case, either a given order is zero, or the total response is dominated by one order only. In the latter case, the total response (sum of all orders) is substantially identical to said order and the two curves are not distinguishable.

Equation (30) allows us to highlight the general aspects of the Stimulated Raman response of a sample as well as to quantify the impact of a high-intensity-modulation frequency on the observed Raman lineshape, which was the main objective of this paragraph:

- The in-phase signal $SRL_{0}$ is proportional to the imaginary part of $\chi^{3}\left(\Omega \right)$, which is what one expects from conventional SRS theory [47]. The spectral shape of this term faithfully reproduces the shape that one could observe for the same transition and for the same thermodynamic conditions in a spontaneous Raman regime.
- The first-order perturbation ($SRL_{1}$) to the in-phase signal is proportional to the real part of the first-order derivative of $\chi^{3}\left(\Omega \right)$, which is an even function of Ω since $Re\left\{\chi^{3}\left(\Omega \right)\right\}$ has a dispersive shape (odd function). However, this contribution nullifies for a proper demodulation phase ($∆=\varphi_{b0}$); therefore, it may affect the lineshape only in the presence of a phase demodulation error, as shown below in Figure 3.
- The second-order perturbation $SRL_{2}$ adds up to $SRL_{0}$ by an amount that depends on: (i) the specific modulation waveform, due to the different $\alpha_{i}\alpha_{j}^{*}$ combinations that enter the expression of $b_{0}$ and $b_{2}$. In our experimental case, the modulation waveform was responsible for $\frac{\left|b_{0}\right|}{\left|b_{2}\right|}\approx 2$ (ii) the weight of the second-order term $\frac{1}{2}\frac{{𝜕}^{2}\chi^{3}}{𝜕\Omega^{2}}\omega_{m}^{2}$ as compared to the leading term $\chi^{3}\left(\Omega \right)$: assuming, as an example, a Lorenztian profile with FWHM=Γ=250 MHz and a modulation frequency $f_{m}=\omega_{m}/2\pi =10\;MHz\;$(which is close to our experimental conditions for the Q(1) 1-0 H_2_ line at pressure > 1 atm), the ratio, at peak, between the two orders is $4{\left(\frac{f_{m}}{\Gamma }\right)}^{2}=0.0064$. Combining the two effects, the perturbation is, thus, in our case, at the 0.3% level. It is worth noting that with a five-times-smaller modulation frequency or a five-times-larger profile (as it happens in H_2_ at low pressure where the linewidth is dominated by Doppler broadening), the distortion is reduced to below 10^−4^.
- The $b_{0}$ and $b_{2}$ coefficients in Equation (30) can be calculated through Equation (18) from the Fourier coefficients of a series expansion of the modulation waveform. Since the latter can be easily measured with a photodetector and an oscilloscope at the output of the modulator, the signal of interest $SRL_{0}$ and its second-order distortion $SRL_{2}$ can be fully quantified. This enables us to extract spectroscopic parameters of high metrological quality from the fitting of experimental spectra, even in the presence of modulation-induced artifacts.

## 3. Results

Figure 3a,c quantify, through Equation (29), the in-phase signals that come into play when probing a Lorentzian Raman transition with FWHM=250 MHz with a modulation waveform at 10 MHz, in the case of an optimal demodulation phase ($∆=\varphi_{b0}$) (a panel) and of the demodulation phase error by 0.05 rad (c panel). The modulation waveform $I_{s}\left(t\right)$ chosen for the simulations in Figure 3 is a piecewise function composed of cubic polynomials (to account for intensity changes) and constants (to represent the steady-intensity parts) that matches our experimental case. In both cases, the in-phase SRL signal remains distorted by no more than three parts over 10^3^ at the centre of the transition (i.e., at zero-frequency detuning between pump and Stokes), but the actual distortion profile depends on the demodulation phase, as an incorrect phase leads to the onset of a first-order distortion $SRL_{1}$ (Figure 3c).

Before further commenting on Figure 3, it is of interest to calculate the quadrature signal given by the lock-in amplifier, setting ${∆=\varphi }_{b0}+\frac{\pi }{2}$ in Equation (29):

(31) $$\begin{array}{c} I_{d0⊥}=2\left|∆E_{0}\right|E_{p}sen\left(\varphi_{\chi }\right)cos\left(\varphi_{b0}-\varphi_{b0}-\frac{\pi }{2}\right)=0\\ I_{d1⊥}=2\left|∆E_{1}\right|E_{p}cos\left(\varphi_{\chi }\right)sen\left(\varphi_{b1}-\varphi_{b0}-\frac{\pi }{2}\right)=2\left|∆E_{1}\right|E_{p}cos\left(\varphi_{\chi }\right)sen\left(\pi -\frac{\pi }{2}\right)=2\left|∆E_{1}\right|E_{p}cos\left(\varphi_{\chi }\right)\\ I_{d2⊥}=2\left|∆E_{2}\right|E_{p}sen\left(\varphi_{\chi \prime \prime }\right)cos\left(\varphi_{b2}-\varphi_{b0}-\frac{\pi }{2}\right)=2\left|∆E_{2}\right|E_{p}sen\left(\varphi_{\chi \prime \prime }\right)sen\left(\varphi_{b2}-\varphi_{b0}\right) \end{array}$$

For optimal demodulation, this reduces to:

(32) $$\begin{array}{c} SRL_{0⊥}=0\\ SRL_{1⊥}=\frac{3}{2}\frac{\omega_{p}}{{{\varepsilon_{0}c}^{2}n}_{p}n_{s}}\langle I_{s}\rangle LRe\left\{\frac{𝜕\chi^{3}}{𝜕\Omega }\right\}\\ SRL_{2⊥}=\frac{3}{2}\frac{\omega_{p}}{{{\varepsilon_{0}c}^{2}n}_{p}n_{s}}\langle I_{s}\rangle L\left|b_{2}\right|Im\left\{\frac{{𝜕}^{2}\chi^{3}}{𝜕\Omega^{2}}\right\}sen\left(\varphi_{b2}-\varphi_{b0}\right) \end{array}$$

We can observe the following:

- There is no zero-order contribution to the quadrature signal if one chooses the proper demodulation phase, i.e., $SRL_{0⊥}=0$.
- The quadrature signal is dominated by the first-order perturbation, precisely by the real part of $\frac{𝜕\chi^{3}}{𝜕\Omega }$, or equivalently by the first-order derivative of $Re\left\{\chi^{3}\right\}$. Since $Re\left\{\chi^{3}\right\}$ has a dispersive shape, the quadrature signal $SRL_{1⊥}$ presents a symmetric spectral response, with a distinguishable peak at the centre of the transition. This is clearly visible in Figure 3b,d, which give the quadrature signal for the correct and wrong demodulation phase, respectively.
- The second-order perturbation $SRL_{2⊥}$ remains negligible for typical modulation waveforms with respect to the first-order term and as long as the demodulation-phase error is relatively small, as in the case of Figure 3d.
- If the demodulation phase is offset from the optimal value, all $SRL_{i⊥}$ orders come into play in the quadrature signal; the $SRL_{0⊥}$ term, in particular, may become prominent, and this can lead, as shown in Figure 3d, to significant changes in the spectral shape of the quadrature signal with the appearance of a double peak. This peculiar shape has the advantage of providing the experimentalist an indication of a wrong demodulation phase, helping to restore the optimal detection conditions.

The above analysis illustrates, first of all, how to select the correct demodulation phase to minimize spectral distortions caused by modulation, as the correct phase corresponds to having just one peak in the quadrature signal. Secondly, it allows one to quantify these distortions once the proper demodulation phase is selected and the intensity modulation waveform is measured. The computed distortions can then be included in the lineshape model adopted to fit the experimental SRS spectra or simply removed from the measured spectra before they are fitted, in such a way to eliminate any systematic error in the retrieved spectroscopic parameters.

## 4. Discussion

**Figure 4:**
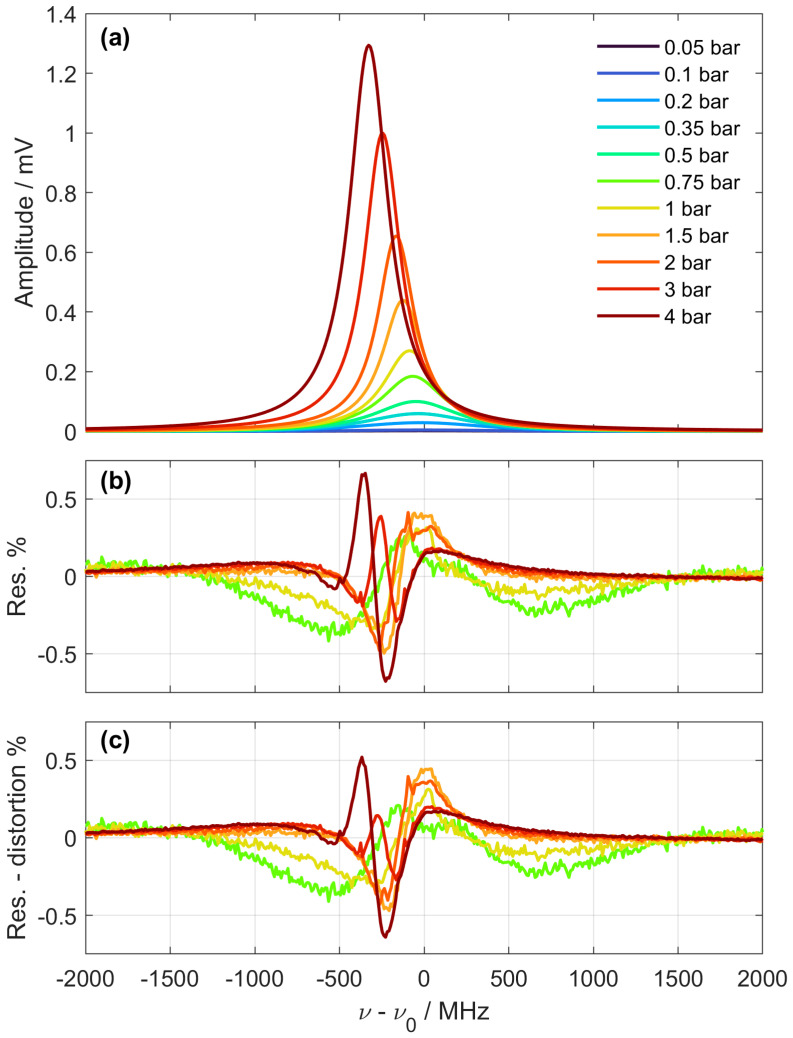
(**a**) Measured SRS spectra of the Q(1) 1-0 line of H2 at different pressures. (**b**) Residuals obtained for the above spectra from a global fitting performed over 9 spectral datasets at different pressures, from 0.05 to 4 bar, using a beta-corrected Harmann Tran profile [49] (see [43] for details). (**c**) Residuals obtained by including in the fitting model the second order distortion computed in this paper through Equation (30).

In this paragraph, we discuss the implications of the above analysis in the interpretation of the metrological results recently obtained by our group on the fundamental Q(1) line of H_2_, by means of a comb-calibrated SRL spectrometer [43]. Spectral measurements were conducted at nine different pressure levels, ranging from 0.05 to 4 bar, maintaining a constant temperature of 303.1 K. A typical set of spectra is shown in Figure 4a. The lineshape and width vary significantly across the pressure range; notably, at high pressure, the spectra become narrower and tend to exhibit a Lorentzian lineshape, whereas at lower pressures, the lineshape becomes broader and Gaussian. This change is due to the Dicke effect, which narrows the velocity distribution of gas molecules and reduces the Doppler profile at higher collision rates. This effect is prominent around 2 bar, nearly eliminating Doppler broadening and resulting in a minimum spectral width of 260 MHz. The SRL spectra were modelled with a β-corrected Hartmann–Tran profile (βHTP) [49], which is an optimized profile for H_2_ isotopologues that accounts, aside from the Dicke effect, for speed-dependent collisional effects and their interplay with velocity-changing collisions. The multi-pressure spectral dataset was globally fitted by bHTP in order to retrieve the line-centre frequency, retaining some collisional parameters fixed against ab-initio calculated values [50] to reduce the correlation between fitted parameters (see [10] for details). The residuals from the global fitting are shown in Figure 4b in normalized units. Regardless of the pressure, the residuals present structured profiles up to 1% of the line maximum. We can, at this point, quantify how much of this structure is due to intensity modulation effects, using Equation (30) to numerically compute the second-order distortion ($SRL_{2}$) of the fitted profile when the intensity modulation of the Stokes field is performed at 10 MHz. The modified residuals, obtained upon subtraction of this distortion, are shown in Figure 4c. At the higher pressures of 2 and 3 bar, where the second-order derivative is maximum because of the line narrowing, the inclusion of this distortion is definitely observable, at the 0.2% level, and beneficially reduces the deviation of residuals from zero. This is a signature that modulation effects must be taken into account for an accurate fitting. On the other hand, a noticeable deviation remains mostly due to the incapacity of βHTP to model H_2_ profiles across such a large pressure range. If one considers low-pressure spectra, which are almost four-times larger because of the prevailing Doppler-broadening contribution, modulation-induced distortions are damped by a factor of ~16, due their quadratic dependence on the ratio between spectral width and modulation frequency. Therefore, for metrological purposes on rovibrational lines, where the goal is to infer the transition line centre mostly from low-pressure spectra, the impact of the developed amplitude-modulation formalism on the uncertainty budget remains poor, because of the dominant error induced by the collisional lineshape model. Conversely, on purely rotational lines lying at frequencies in the few hundreds of wavenumbers—as opposed to rovibrational ones lying at thousands of wavenumbers—the Doppler contributions would be significantly reduced, and the impact of the formalism presented in this study would be much more significant.

**Figure 5:**
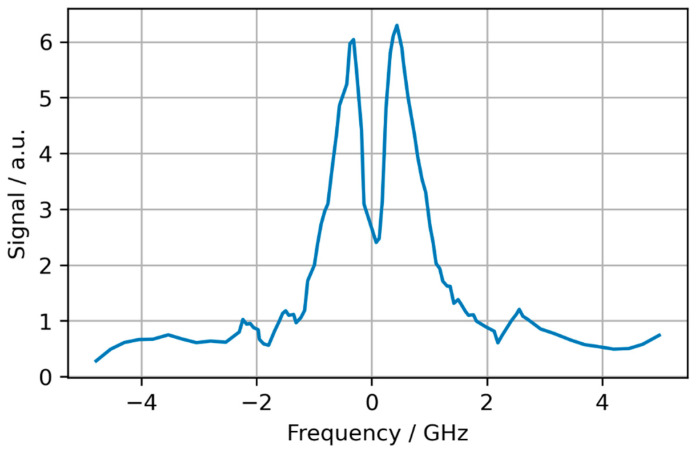
Quadrature signal adapted from [44]: this shape can be explained with a slightly wrong demodulation phase (see Figure 3d).

As a final element of discussion, we notice that our analysis provides an explanation, for the first time, of an unexpected behaviour of the quadrature signal observed in 1991 by A. D May and co-workers [44] using a similar spectrometer. This signal is reproduced in Figure 5 and exhibits a double-peak shape that is a clear signature, in the light of Figure 3b,d, of a slightly wrong demodulation phase. Subtly, the experimentalist that would like to minimize the quadrature signal is easily brought to select such a wrong demodulation phase, as it leads, due to the opposite signs of $SRL_{0⊥}$ and $SRL_{1⊥}$ in Equation (32), to a two-lobe spectrum, whose peak (Figure 3d) is half that with a correct phase (Figure 3b). In turn, a wrong demodulation phase affects the principal signal, namely the in-phase signal, as it introduces a first-order perturbation (Equation (30)).

## 5. Conclusions

We presented a comprehensive analysis of the perturbation produced by an intensity-modulated Stokes field on the spectral lineshape observed in a Stimulated Raman Loss spectrometer. The extension of the analysis and of the derived formula to the case of Stimulated Raman Gain is straightforward. As a rule of thumb, the first-order perturbation induced by intensity modulation can be suppressed by the choice of a proper demodulation phase, while the second-order contribution weighs nearly two-times the squared ratio between the modulation frequency and the linewidth for nearly Lorentzian lines. In our metrological investigation of the fundamental rovibrational band H_2_, the distortion of the SRS response is at the 0.2–0.3% level at pressures larger than 1 bar. As this is smaller than the deviation between experimental and fitted spectra introduced in the βHTP model adopted of the fitting, no significant improvement in the uncertainty budget can, at present, be inferred by the analytical tools developed in this study. Prospectively, they can be better leveraged when more accurate lineshape models are available or simply through the observation of narrower spectra. This will be the case in the forthcoming spectroscopic investigation of rotational H_2_ lines, whose low-pressure profiles are as narrow as 150 MHz and characterized by easier-to-model Doppler profiles. Finally, we point out that the formalism here developed offers the chance to compute any harmonics of the modulation frequency and is susceptible to being extended to other nonlinear spectroscopy settings operated under amplitude or frequency modulation.

## Data Availability

Datasets are available upon request to the corresponding author.

## Supplementary Materials

The following supporting information can be downloaded at: https://www.mdpi.com/article/10.3390/s24216990/s1, File S1: An analysis of the phase matching condition in an SRS process under amplitude modulation of the Stokes field is there provided.
