# Supplementary material for: Lineshape of Amplitude-Modulated Stimulated Raman Spectra
Source: Sensors (Basel). 2024 Oct 30;24(21):6990. doi: 10.3390/s24216990 (PMC11548216; doi:10.3390/s24216990)
Supplement: Supplementary file 1 [file sensors-24-06990-s001.zip › sensors-3231429-supplementary.pdf]

## Lineshape of amplitude-modulated Stimulated Raman spectra

## *Supplementary material*

### Phase matching condition in an intensity modulated SRS process

The absence of a phase-matching condition in an SRS process is a direct consequence of the typically adopted monochromatic assumption for the interacting pump and Stokes fields. Since this assumption fails in the presence of sidebands for the driving field, it is worth determining which phase-matching term intervenes in our experimental case and how much it affects the SRS response in an interaction over quite a long path of 30 m. Let us focus, without loss of generality, on one of the many possible interactions highlighted in Eq. (13) that lead to a nonlinear polarization wave oscillating at $\omega_p + \omega_m$:

$$\tilde{P}_{NL}(\omega_p + \omega_m) = \frac{6}{8}\epsilon_0 \chi^3(-[\omega_p + \omega_m], \omega_p, \omega_s, -[\omega_s - \omega_m]) E_p E_s E_{s-1}^* e^{-i(\omega_p+\omega_m)t} +$$

The wavenumber associated to this nonlinear polarization, as expanded in series, is given by:

$$k_{NL} = k(\omega_p) + k(\omega_s) - k(\omega_s - \omega_m) = k(\omega_p) + k(\omega_s) - \left[k(\omega_s) + \left.\frac{dk}{d\omega}\right|_{\omega_s}(-\omega_m)\right]$$
$$= k(\omega_p) + \left.\frac{dk}{d\omega}\right|_{\omega_s}\omega_m$$

whereas the wavenumber of the generated wave at $\omega_p + \omega_m$ is:

$$k(\omega_p + \omega_m) = k(\omega_p) + \left.\frac{dk}{d\omega}\right|_{\omega_p}\omega_m$$

The dephasing between driving and forced waves upon a propagation over a length $L$ is thus:

$$\Delta kL = [k(\omega_p + \omega_m) - k_{NL}]L = \left[\left.\frac{dk}{d\omega}\right|_{\omega_p} - \left.\frac{dk}{d\omega}\right|_{\omega_s}\right]\omega_m L = \left(\frac{1}{v_{gp}} - \frac{1}{v_{gs}}\right)\omega_m L = GDM\omega_m L$$

It depends on the group delay mismatch per unit length ($GDM$) between pump and Stokes waves. As an example, at a pressure of 1 atm in air where $GDM = 1.5 \cdot 10^{-5}\, ps/mm$, a dephasing as small as $\Delta kL$ = $2 \cdot 10^{-5} rad$ is readily calculated in our experimental conditions, where $\omega_m = 2\pi \cdot 10MHz$ and $L = 30m$. This excludes the need for any phase-matching related caveat.
